# Feasibility of Renal Blood Flow Measurement Using ^64^Cu-ATSM PET/MRI: A Quantitative PET and MRI Study

**DOI:** 10.3390/diagnostics13101685

**Published:** 2023-05-10

**Authors:** Yudai Nishikawa, Naoki Takahashi, Sho Nishikawa, Yuki Shimamoto, Kazuhisa Nishimori, Mamiko Kobayashi, Hideki Kimura, Tetsuya Tsujikawa, Kenji Kasuno, Tetsuya Mori, Yasushi Kiyono, Hidehiko Okazawa, Masayuki Iwano

**Affiliations:** 1 Department of Nephrology, Faculty of Medical Sciences, University of Fukui, Fukui 910-1193, Japan; 2 Biomedical Imaging Research Center, University of Fukui, Fukui 910-1193, Japan; 3 Department of Radiology, Faculty of Medical Sciences, University of Fukui, Fukui 910-1193, Japan

**Keywords:** renal blood flow, ^64^Cu-ATSM, arterial spin labeling, positron emission tomography/magnetic resonance imaging, creatinine, cystatin C

## Abstract

This study aimed to evaluate the renal blood flow (RBF) in patients with chronic kidney disease (CKD) using ^64^Cu(II)-diacetyl-bis(4-methylthiosemicarbazonate) (^64^Cu-ATSM) for positron emission tomography (PET)/magnetic resonance imaging (MRI). We included five healthy controls (HCs) and ten patients with CKD. The estimated glomerular filtration rate (eGFR) was calculated from the serum creatinine (cr) and cystatin C (cys) levels. The estimated RBF (eRBF) was calculated using the eGFR, hematocrit, and filtration fraction. A single dose of ^64^Cu-ATSM (300–400 MBq) was administered for RBF evaluation, and a 40 min dynamic PET scan was performed with simultaneous arterial spin labeling (ASL) imaging. PET-RBF images were obtained from the dynamic PET images at 3 min after injection using the image-derived input function method. The mean eRBF values calculated from various eGFR values differed significantly between the patients and HCs; both groups also differed significantly in terms of the RBF values (mL/min/100 g) measured using PET (151 ± 20 vs. 124 ± 22, *p* < 0.05) and ASL-MRI (172 ± 38 vs. 125 ± 30, *p* < 0.001). The ASL-MRI-RBF was positively correlated with the eRBFcr-cys (r = 0.858, *p* < 0.001). The PET-RBF was positively correlated with the eRBFcr-cys (r = 0.893, *p* < 0.001). The ASL-RBF was positively correlated with the PET-RBF (r = 0.849, *p* < 0.001). ^64^Cu-ATSM PET/MRI demonstrated the reliability of PET-RBF and ASL-RBF by comparing them with eRBF. This is the first study to demonstrate that ^64^Cu-ATSM-PET is useful for assessing the RBF and is well correlated with ASL-MRI.

## 1. Introduction

To understand the pathophysiology of chronic kidney disease (CKD), renal blood flow (RBF) should be evaluated. RBF can be determined by the clearance method using para-aminohippuric acid and estimated blood data. However, this method is now rarely performed in clinical practice because of the need for repeated blood and urine collection, high patient stress, and lack of rapidity. Furthermore, it cannot be used to measure separate renal functions. The estimated RBF (eRBF) is the most widely used parameter for evaluating renal function; it is calculated from the estimated glomerular filtration rate (eGFR) using hematocrit (Ht) and filtration fraction (FF). However, it is only an estimate of the total body clearance and not of the individual kidney function. Nuclear medicine methods are also commonly used for RBF measurement during renal function assessment; they enable the evaluation of separate renal functions. However, due to the poor resolution and attenuation of radioactivity in the body, the quantitative values obtained from these methods are not stable.

To overcome these limitations, RBF evaluation with magnetic resonance imaging (MRI)-arterial spin labeling (ASL), using blood water as an endogenous tracer, was recently investigated and shown to be reasonable [1,2,3]. However, no studies on the use of positron emission tomography (PET) for RBF measurement are available; this is probably because nuclear medicine modalities other than PET are generally used in clinical practice. However, the image quality and quantitative accuracy obtained by PET are better than those obtained by other nuclear medicine modalities using single-photon tracers. Cu (II)-diacetyl-bis(4-methylthiosemicarbazonate) (Cu-ATSM) labeled with radioactive copper is a tracer developed for PET hypoxia imaging [4,5,6,7]. Recently, it has been used for in vivo imaging of oxidative stress and blood flow [6,8,9,10,11]. In this study, we estimated the renal function in patients with CKD using these two modalities, ^64^Cu-ATSM PET/MRI. MRI and PET image acquisition were performed simultaneously to measure the RBF of each kidney using ASL and PET-RBF images. Furthermore, we evaluated the reliability of ^64^Cu-ATSM PET/MRI by comparing eRBF (obtained using clinical parameters) with the measured RBF. The tracer must reach the target organ appropriately during PET. We have accordingly proposed a PET-based method for accurate RBF assessment, and have compared it with other modalities before understanding the oxidative stress involved in CKD imaging in future studies.

## 2. Materials and Methods

### 2.1. Participants

Ten patients with CKD who visited our outpatient clinic for regular checkups participated in this study. These comprised five patients with diabetic kidney disease (DKD), two with nephrosclerosis (NS), two with IgA glomerulonephritis (IgAGN), and one with a non-IgAGN case. Five age-matched healthy controls (HCs) were also included. All participants provided full informed consent, and the study was approved by the Research Ethics Committee of the University of Fukui Hospital (20170053) and complied with the Declaration of Helsinki. The Division of Clinical Laboratories, Fukui University Hospital, examined the blood chemistry, including the serum creatinine (cr) and cystatin C (cys) levels (Fukui, Japan). The body height and body weight were measured before PET. The Ht levels were measured using an automated analyzer.

### 2.2. eRBF of the Kidney

The kidney eRBF was calculated from the serum cr and cys levels as follows. First, the eGFR was calculated from the serum cr and cys levels using the following equations [12,13,14]: 

eGFRcr (mL/min/1.73 m^2^) = 194 × serum cr^−1.094^ × age^−0.287^ (× 0.739 [if female]) 

eGFRcys (mL/min/1.73 m^2^) = (104 × serum cys^−1.019^ × 0.996^age^ [× 0.929 {if female}]) − 8

eGFRcr-cys = 135 × min (serum cr/κ, 1)^α^ × max (serum cr/κ, 1)^−0.544^ × min (serum cys/0.8, 1)^−0.323^ × max (serum cys/0.8, 1)^−0.778^ × 0.9961^Age^ × 0.963 (if female)

Second, the body surface area (BSA) of each participant was calculated using the formula provided by DuBois et al. [15]. Third, BSA-unadjusted eGFR was calculated using the following formula: BSA-unadjusted eGFR (mL/min) = eGFR × (BSA/1.73). Fourth, BSA-unadjusted estimated renal plasma flow (eRPF) was calculated as follows: BSA-unadjusted eRPF (mL/min) = (BSA-unadjusted eGFR)/FF. The FF values for HCs and patients with primary disease were obtained from previous reports (controls: 0.20 [16], DKD with proteinuria < 0.3 g/day: 0.224 [17], DKD with proteinuria > 0.5 g/day: 0.21 [18], IgAGN and non-IgAGN: 0.22 [19], and NS: 0.18 [20]). Fifth, the BSA-unadjusted eRBF was then calculated from the eRPF using Ht: BSA-unadjusted eRBF (mL/min) = BSA-unadjusted eRPF (100/[100 − Ht {in %}]).

### 2.3. PET/MRI Imaging

In accordance with a previously described procedure [21,22], gold discs (diameter: 25 mm, depth: 2 mm; dimpled in the center) were created and electrodeplated with enriched ^64^Ni (>98%) to produce ^64^Cu. An RDS Eclipse biomedical cyclotron (Siemens, Göteborg, Sweden) was used to bombard the gold discs with 11 MeV protons. ^64^Cu was separated using a previously described process, and ^64^Cu-ATSM was obtained by stirring a ^64^Cu-glycine solution with ATSM [21].

All participants underwent ^64^Cu-ATSM PET/MRI with a whole-body scanner (Signa PET/MR ver. 26, GE Healthcare, Milwaukee, WI, USA). The scanner allows the simultaneous acquisition of PET data in 89 image slices (slice thickness: 2.78 mm) in three dimensions [23,24]. According to performance tests, the transaxial intrinsic resolution of the PET images was between 4.2 mm and 4.3 mm (full width at half maximum). Before ^64^Cu-ATSM PET/MRI was performed, the PET/MRI scanner was calibrated with a dose calibrator (CRC-12, Capintec Inc., Florham Park, NJ, USA) using a pool phantom and an 18F solution, according to the manufacturer’s instructions [23,24].

After a bolus injection of ^64^Cu-ATSM (300–400 MBq [8.1–10.8 mCi]) into the antecubital vein, a 40 min list-mode 3D PET scan was initiated using the time-of-flight acquisition mode. During the PET scan, several MRI sequences, including LAVA-FLEX (GE Healthcare) T1WI, T2WI, ASL, and DIXON, for PET attenuation correction (AC) imaging data were acquired [23,24]. During the ASL scan, the participants held their breath for approximately 50 s to avoid abdominal movements. Details of the ASL sequence are provided below.

Using the 3D-ordered subset expectation maximization (OSEM) method and the point spread function modeling algorithm, dynamic PET image data were reconstructed from the list-mode PET and MR-AC data in 35 frames (12 × 5 s, 6 × 10 s, 3 × 20 s, 4 × 30 s, 5 × 60 s, 4 × 5 min, and 1 × 10 min). The following OSEM parameter set was utilized for the reconstruction of both PET images: subsets, 16; iterations, 2; transaxial post-Gaussian filter cutoff, 4 mm in a 384 mm field of view (FOV); and pixel size, 3 × 3 mm^2^. A static PET image averaging 15–40 min post-injection data was used to assess ^64^Cu-ATSM uptake in the kidney. The following equation was used to convert the average PET image to a standardized uptake value (SUV) image using each participant’s injection dose and body weight: SUV = (tissue concentration)/([injection dose] × [body weight]).

### 2.4. RBF from ^64^Cu-ATSM-PET

The RBF image was calculated based on dynamic PET data from the first 3 min and a single-tissue compartment model using the following equation:

(1) $$M\left(t\right)=K_{1}C_{a}\left(t\right)⊗e{\;}^{-k_{2}t}+V_{0}C_{a}\left(t\right)$$

Here, M(t) is the radioactivity concentration in the renal tissue (as determined by PET), and C_a_(t) is the arterial time–radioactivity curve (TAC) measured from the abdominal aorta using dynamic PET data and the image-derived input function (IDIF) method [25]. The rate constants for influx and efflux of the tracer were K_1_ and k_2_, respectively, and the vascular volume of the arterioles to the arterial capillaries is denoted by V_0_. The symbol ⊗ represents the convolution process. The following equation uses three different *w*_i_ values (i = 1–3) as weights, which can be obtained from Equation (1).

(2) $$\overset{T}{\underset{0}{\int }}w_{i}\left(t\right)M\left(t\right)dt=K_{1}\overset{T}{\underset{0}{\int }}w_{i}\left(t\right)C_{a}\left(t\right)⊗e{\;}^{-k_{2}t}dt+V_{0}\overset{T}{\underset{0}{\int }}w_{i}\left(t\right)C_{a}\left(t\right)dt$$

Equation (2) can be solved for K_1_ using a look-up table with the variable k_2_, which is the TAC of renal tissue from the PET data. Three image slices were used to set up circular regions of interest (ROIs; diameter: 10 mm) in the abdominal aorta at the level of the renal artery to estimate the arterial input function (C_a_). The mean ROI values for each frame were calculated as the arterial radioactivity at the mid-frame time [23]. Because the extraction (E) of ^64^Cu-ATSM into renal tissue is considered to be almost 1.0, we assumed that K_1_ (= RBF × E) is very close to RBF; thus, we used the K_1_ image for PET-RBF evaluation. The weighted integral approach for perfusion PET studies is described elsewhere [25,26].

### 2.5. RBF from ASL-MRI

The scout images were scanned with a gradient echo sequence in three planes at the center of each kidney to determine the location of the ASL perfusion images. Coronal T2-weighted imaging was performed for anatomical and volume evaluations of the entire kidney using a single-shot fast spin-echo sequence with the following parameters: TR, 3800 ms; TE, 80 ms; image matrix, 352 × 224 slice thickness, 4.0 mm; interval, 0 mm; flip angle, 90°; bandwidth, 83.33 kHz; and FOV, 36 cm. The participants held their breath for approximately 50 s to allow ASL image acquisition during the MRI acquisition process. The precise parameters and settings for pseudo-continuous ASL (GE’s product version) are described elsewhere [27]; background suppression and 3D spiral fast spin-echo acquisition were performed with the following scanning parameters: FOV, 360 mm; matrix size, 128 × 128; in-plane resolution, 2.8 mm; slice thickness, 4.0 mm; and single post-labeling delay, 1.0 s [3]. The labeling slab was automatically positioned at a level of 2 cm above the scan range. The number of excitations for the acquisition was 1, and the total scan duration was 50 s. An approximate proton density-weighted image was acquired for blood flow quantification using the same acquisition parameters. The model presented by Alsop et al. was used to calculate the RBF [23,28,29], with the inclusion of a term for the labeling duration.

### 2.6. Statistical Analysis

For quantitative analysis of the RBF, multiple circular ROIs (diameter: 10 mm) were placed using several slices of the bilateral renal cortex on LAVA-FLEX MRI images to identify the renal cortical tissue. The ROIs were applied to PET-RBF and ASL-RBF images at the same locations. The average value of 20 ROIs was calculated to obtain the RBF values for each kidney. On a workstation (AW4.6, GE Healthcare, Milwaukee, WI, USA), renal volumes (cm^3^) were determined using image analysis software. Bilateral kidney areas were determined using each slice of the T2-weighted axial image that covered the entire kidney. For each kidney, the kidney area was multiplied by the slice thickness and summed [3]. The mean RBF values (mL/min/100 g) obtained from the PET and MRI images were corrected for the RBF in each kidney (mL/min) using the individual renal volume. The eRBF per kidney, determined using the eGFR, and RBF values obtained from the PET/MRI images were evaluated using a one-way analysis of variance for multiple comparisons. A post hoc test was performed using the Fisher least significant difference method. A Pearson’s linear regression analysis was performed to observe the relationships between the parameters, and the correlation coefficient was determined for each regression. Values with *p* < 0.05 were considered significant. The agreement between ASL-RBF and PET-RBF was evaluated using a Bland–Altman analysis. All analyses were performed with SigmaPlot 15 (SystatSoftware, Inc., San Jose, CA, USA) and EZR v1.54 (Jichi Medical University Saitama Medical Center, Saitama, Japan).

## 3. Results

### 3.1. RBF Values from the Clinical Parameters and ^64^Cu-ATSM PET/MRI

The RBF values (mean ± standard deviation [SD]) calculated from the clinical parameters (i.e., eRBF) and ^64^Cu-ATSM PET/MRI are presented in Table 1; data for both HCs and patients with CKD are provided.

The SUV values from ^64^Cu-ATSM-PET are also included in Table 1. The eRBF (from clinical parameters) and RBF (from PET/MRI) were significantly lower in patients with CKD than in HCs (*p* < 0.05). However, the ^64^Cu-ATSM-PET SUV did not differ significantly between the two groups.

### 3.2. Correlation between the eRBFs and the RBF from PET/MRI

The upper half of Table 2 summarizes the correlation between the eRBF and RBF values from the images.

**Figure 1:**
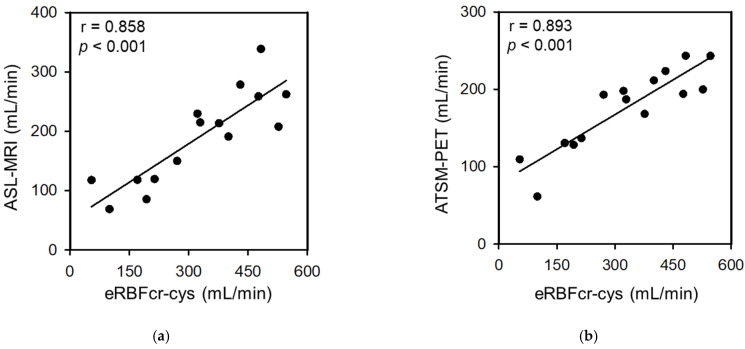
Correlation between RBF (measured by PET/MRI) and eRBFcr-cys (measured from serum parameters). Scatter plot of eRBF obtained by clinical parameters and RBF obtained by ASL-MRI and ^64^Cu-ATSM-PET. The eRBF was positively correlated with (**a**) ASL-MRI (r = 0.858, *p* < 0.001) and (**b**) ^64^Cu-ATSM-PET (r = 0.893, *p* < 0.001). ASL, arterial spin labeling; MRI, magnetic resonance imaging; PET, positron emission tomography; eRBF, estimated renal blood flow; Cu-ATSM, Cu (II)-diacetyl-bis(4-methylthiosemicarbazonate; cys, cystatin C; cr, creatinine.

As shown in Figure 1a, the RBF obtained from ASL-MRI correlated with the eRBFcr (r = 0.823, *p* < 0.001), eRBFcys (r = 0.829, *p* < 0.001), and eRBFcr-cys (r = 0.858, *p* < 0.001). 

As shown in Figure 1b, the RBF (measured with ^64^Cu-ATSM-PET) was correlated with the eRBFcr (r = 0.871, *p* < 0.001), eRBFcys (r = 0.866, *p* < 0.001), and eRBFcr-cys (r = 0.893, *p* < 0.001). 

### 3.3. Correlation between RBF Values from ASL-MRI and ^64^Cu-ATSM-PET

**Figure 2:**
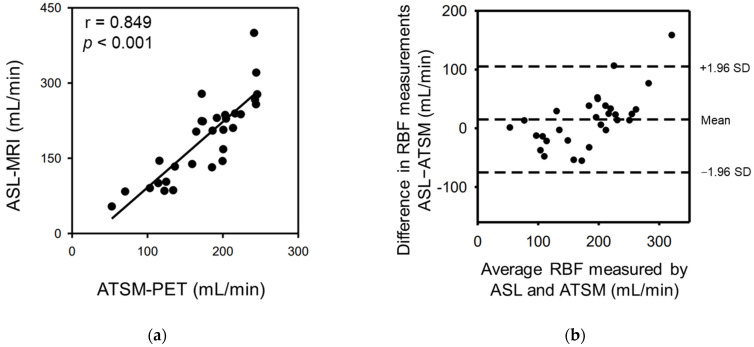
Correlation between the ASL-MRI-RBF and ^64^Cu-ATSM-PET-RBF and Bland–Altman analysis. RBF scatterplots obtained from ASL-MRI and ^64^Cu-ATSM-PET imaging (**a**) (r = 0.849, *p* < 0.001). Bland–Altman plots of RBF obtained by ASL-MRI and ^64^Cu-ATSM-PET imaging (**b**). The dotted lines denote the mean and 1.96 SD bounds. ASL, arterial spin labeling; MRI, magnetic resonance imaging; PET, positron emission tomography; RBF, renal blood flow; Cu-ATSM, Cu (II)-diacetyl-bis(4-methylthiosemicarbazonate; SD, standard deviation.

The RBF measured using ASL-MRI was well correlated with that measured using ^64^Cu-ATSM-PET (r = 0.849, *p* < 0.001; Figure 2a).

The Bland–Altman analysis revealed good agreement between the RBF measured by ASL-MRI and that measured by ^64^Cu-ATSM-PET (bias = 7.9, SD = 33.8; Figure 2b).

### 3.4. Correlation between the Adjusted Body Surface Area eRBFs and RBF from the Images

The lower half of Table 2 summarizes the correlation between the BSA-adjusted eRBF and RBF from the images. The RBF measured using ASL-MRI was correlated with the BSA-adjusted eRBFcr (r = 0.786, *p* < 0.001), eRBFcys (r = 0.823, *p* < 0.001), and eRBFcr-cys (r = 0.834, *p* < 0.001). The RBF measured using ^64^Cu-ATSM-PET was also correlated with the BSA-adjusted eRBFcr (r = 0.846, *p* < 0.001), eRBFcys (r = 0.868, *p* < 0.001), and eRBFcr-cys (r = 0.879, *p* < 0.001). 

### 3.5. Representative Images

**Figure 3:**
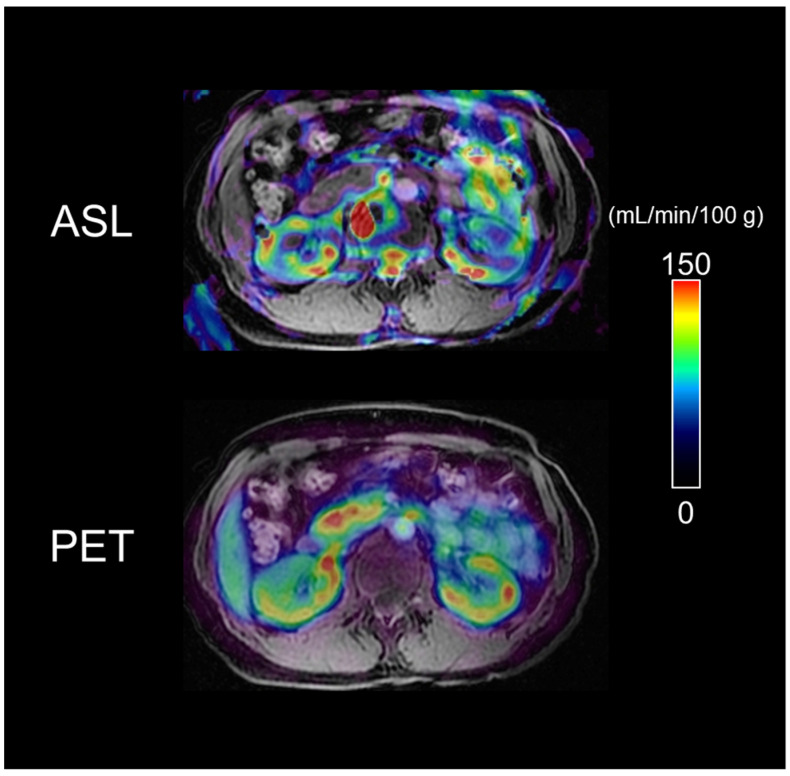
Representative ASL-MRI and ^64^Cu-ATSM-PET images for RBF evaluation in HCs. Both ASL-MRI and ^64^Cu-ATSM-PET allowed visual evaluation of the RBF in the kidney parenchyma. The colour of this scale bar indicates blood flow (mL/min/100 g), with red indicating more blood flow. ASL, arterial spin labeling; MRI, magnetic resonance imaging; PET, positron emission tomography; RBF, renal blood flow; Cu-ATSM, Cu (II)-diacetyl-bis(4-methylthiosemicarbazonate; HC, healthy control.

Representative ASL-MRI and ^64^Cu-ATSM-PET images are shown in Figure 3 (^64^Cu-ATSM images of all participants are presented in Supplementary Figure S1). 

The cortical RBF was heterogeneous in ASL-MRI and relatively homogeneous in ^64^Cu-ATSM-PET.

## 4. Discussion

In this study, quantitative RBF measurement with PET using ^64^Cu-ATSM showed an excellent correlation with the measurement performed using the ASL method, which is considered the only method for the quantitative measurement of blood flow using MRI. The RBF obtained from quantitative PET and MRI imaging was also compared with the eRBF obtained from clinical parameters; excellent correlations were observed between them. These results suggest that these two imaging modalities are feasible for evaluating individual renal function. The PET and MRI images were obtained simultaneously using a PET/MRI scanner; thus, RBF images from the two modalities were obtained simultaneously. To the best of our knowledge, this is the first study to quantify the RBF by each modality on PET/MRI images and is highly reliable owing to simultaneous image acquisition. Our findings are also significant, in that the RBF images from both modalities enabled the evaluation of separate renal functions with high precision. Regional values in the ASL-RBF image are reportedly affected by the post-labeling delay time; however, since the PET-RBF values were calculated from dynamic PET data with an arterial input function, they are thought to have been more accurate than the ASL-RBF values.

For the PET-RBF calculation, a one-tissue compartment two-parameter model was used to avoid the effect of blood pool radioactivity. Because the kidneys contain a substantial volume of blood in the renal cortical parenchyma, the effect of radioactivity from the blood pool should be eliminated from the RBF values. ^64^Cu-ATSM, used in the present study, has been used in clinical studies on oxidative stress and hypoxic tissue imaging of the brain and tumors [5,6,7,8,9,10,11]. These studies have revealed that images from the early phase after tracer injection reflect the blood flow, while images from the later phase (i.e., at or after 10–15 min from the injection) reflect Cu-ATSM retention [5,6]. We calculated the RBF quantitatively using dynamic PET data and the IDIF method (which is considered to provide regional values that are more reliable than the ASL-RBF values, as described above). RBF evaluated using PET/MRI, unlike that evaluated using clearance methods, can accurately assess separate renal functions. Another advantage of ^64^Cu-ATSM RBF is that it can also measure the local RBF of very small ROIs. Evaluation of the oxidative stress in kidneys using ^64^Cu-ATSM is a promising topic for further studies; however, it is worth noting that the SUV values from later-phase images did not differ significantly between patients with CKD and the HCs in this study. The amount of ^64^Cu-ATSM reaching the kidneys may differ between patients with a reduced RBF and the HCs; the RBF values measured with the same tracer should be corrected for this effect to assess the renal oxidative stress accurately.

The present study revealed that the RBF obtained from quantitative PET and MRI images were directly comparable to the eRBF obtained from clinical parameters; excellent correlations were noted between them. The RBF measured by ASL has often been evaluated in correlation with the eGFR [30,31]; a significant correlation has been noted between the two. In our study, the correlation coefficient between the ASL-RBF and eGFR was higher than that reported previously (r = 0.74–0.82, *p* < 0.01). Furthermore, the correlation between the ^64^Cu-ATSM-RBF and eGFR was as good as that between the ASL-MRI and eGFR (r > 0.85, *p* < 0.001; Table 3). 

The higher correlation coefficients observed in this study may be due to two reasons. First, the units of the RBF values from PET/MRI were corrected from mL/min/100 g to mL/min using the kidney weight estimated by volumetry of the MRI images. Without correction for the kidney weight, the correlation coefficients were lower than those reported in the present study. Second, we used the latest equation by Inker et al. to obtain the eGFR in this study [14]. eGFRs were calculated using three estimation equations (eGFRcr, eGFRcys, and eGFRcr-cys), and the eRBF (eRBFcr, eRBFcys, and eRBFcr-cys) was obtained for each eGFR. All correlations between the PET/MRI RBF and the eGFRs and eRBFs obtained using Inker’s equation showed the best correlation coefficients. Therefore, we believe that the equation by Inker et al. is the most desirable for estimating the eRBF from clinical parameters.

This study had several limitations. First, only 15 cases were analyzed; however, this sample size is sufficient for evaluating whether a quantitative approach that compares HCs and patients with CKD is feasible. Second, MRI scanning for the ASL sequence requires a long breath-holding time. Some participants may have moved slightly during this period, which may have affected the quality of the ASL image obtained. Further studies with more cases are needed for the assessment of the RBF and the evaluation of renal oxidative stress in patients with specific CKDs.

## 5. Conclusions

In conclusion, ^64^Cu-ATSM PET/MRI demonstrated the reliability of PET-RBF and ASL-RBF by comparing them with the eRBF calculated from the eGFR. ^64^Cu-ATSM-PET is useful for assessing the RBF and has a good correlation with ASL-MRI.

## Figures and Tables

**Table 1:** Comparison of the eRBF (from clinical parameters), RBF (from PET/MRI), and ^64^Cu-ATSM SUV between HCs and patients with CKD.

	HCs	CKD	*p*
Age	56.2 ± 3.7	63.3 ± 9.0	0.12
eRBF (mL/min)			
eRBFcr	295.6 ± 30.9	174.9 ± 82.7	<0.05
eRBFcys	485.4 ± 92.1	225.5 ± 105.5	<0.001
eRBFcr-cys	486.5 ± 56.4	246.0 ± 121.2	<0.001
RBF (mL/min)			
^64^Cu-ATSM-PET (*n* = 30)	218.5 ± 26.0	153.7 ± 49.3	<0.001
ASL-MRI (*n* = 30)	251.7 ± 66.2	159.6 ± 71.0	<0.01
RBF (mL/min/100 g)			
^64^Cu-ATSM-PET (*n* = 30)	151.0 ± 20.2	123.5 ± 21.5	<0.05
ASL-MRI (*n* = 30)	172.2 ± 38.3	124.8 ± 29.7	<0.001
^64^Cu-ATSM-PET SUV	3.2 ± 1.0	3.1 ± 0.9	0.89

HCs, healthy controls; CKD, chronic kidney disease; eRBF, estimated renal blood flow; cr, creatinine; cys, cystatin C; ^64^Cu-ATSM, ^64^Cu-diacetyl-bis(4-ethylthiosemicarbazonate); ASL, arterial spin labeling; SUV, standardized uptake value.

**Table 2:** Linear correlations between RBF (measured using ASL-MRI or ^64^Cu-ATSM-PET) and eRBF (calculated using clinical parameters).

		Intercept (95% CI)	Slope (95% CI)	r	*p*
PET/MRI-RBF vs. eRBF (unadjusted BSA) (mL/min)	ASL-RBF vs. eRBFcr	36.6 (−31.8 to 105.1)	0.714 (0.419 to 1.009)	0.823	<0.001
	ASL-RBF vs. eRBFcys	63.9 (6.9 to 120.9)	0.405 (0.241 to 0.569)	0.829	<0.001
	ASL-RBF vs. eRBFcr-cys	49.4 (−6.2 to 105.1)	0.432 (0.277 to 0.587)	0.858	<0.001
	^64^Cu-ATSM-RBF vs. eRBFcr	67.1 (27.6 to 106.5)	0.503 (0.333 to 0.672)	0.871	<0.001
	^64^Cu-ATSM-RBF vs. eRBFcys	87.4 (53.5 to 121.3)	0.281 (0.184 to 0.379)	0.866	<0.001
	^64^Cu-ATSM-RBF vs. eRBFcr-cys	77.7 (45.2 to 110.2)	0.299 (0.209 to 0.390)	0.893	<0.001
PET/MRI-RBF vs. eRBF (adjusted BSA) (mL/min/1.73 m^2^)	ASL-RBF vs. eRBFcr	38.6 (−38.2 to 115.4)	0.722 (0.382 to 1.063)	0.786	<0.001
	ASL-RBF vs. eRBFcys	57.9 (−2.6 to 118.3)	0.437 (0.256 to 0.617)	0.823	<0.001
	ASL-RBF vs. eRBFcr-cys	48.6 (−12.9 to 110.1)	0.446 (0.269 to 0.623)	0.834	<0.001
	^64^Cu-ATSM-RBF vs. eRBFcr	66.5 (22.5 to 110.6)	0.518 (0.322 to 0.713)	0.846	<0.001
	^64^Cu-ATSM-RBF vs. eRBFcys	82.3 (47.1 to 117.5)	0.307 (0.202 to 0.412)	0.868	<0.001
	^64^Cu-ATSM-RBF vs. eRBFcr-cys	75.8 (40.5 to 111.1)	0.313 (0.212 to 0.415)	0.879	<0.001
MRI vs. PET-RBF	ASL-RBF vs. ^64^Cu-ATSM-RBF	−39.5 (−97.3 to 18.3)	1.311 (0.995 to 1.627)	0.849	<0.001

CI, confidence interval; RBF, renal blood flow; eRBF, estimated renal blood flow; ASL, arterial spin labeling; cr, creatinine; cys, cystatin C; Cu-ATSM, Cu(II)-diacetyl-bis(4-methylthiosemicarbazonate); BSA, body surface area.

**Table 3:** Linear correlations between the RBF measured with ASL-MRI or ^64^Cu-ATSM-PET and the eGFR calculated by clinical parameters.

	Intercept (95% CI)	Slope (95% CI)	r	*p*
ASL-RBF vs. eGFRcr	33.2 (−56.6 to 122.9)	3.147 (1.454 to 4.839)	0.744	<0.01
ASL-RBF vs. eGFRcys	48.9 (−18.3 to 116.0)	1.973 (1.113 to 2.832)	0.809	<0.001
ASL-RBF vs. eGFRcr-cys	39.4 (−29.9 to 108.7)	2.007 (1.154 to 2.860)	0.816	<0.001
^64^Cu-ATSM-RBF vs. eGFRcr	55.2 (8.7 to 101.7)	2.405 (1.529 to 3.281)	0.854	<0.001
^64^Cu-ATSM-RBF vs. eGFRcys	72.6 (36.8 to 108.4)	1.432 (0.974 to 1.890)	0.882	<0.001
^64^Cu-ATSM-RBF vs. eGFRcr-cys	64.8 (29.6 to 100.0)	1.470 (1.036 to 1.903)	0.897	<0.001

CI, confidence interval; ASL, arterial spin labeling; RBF, renal blood flow; eGFR, estimated glomerular filtration rate; cr, creatinine; cys, cystatin C; Cu-ATSM, Cu(II)-diacetyl-bis(4-methylthiosemicarbazonate).

## Data Availability

The files/data used to support the findings of this study are available from the corresponding author upon request.

## Supplementary Materials

The following supporting information can be downloaded at: https://www.mdpi.com/article/10.3390/diagnostics13101685/s1, Figure S1: ^64^Cu-ATSM-PET images (RBF) of the early phases of all participants. Patients with CKD have reduced RBF and their kidneys are atrophic.
