# Supplementary material for: Feasibility of Renal Blood Flow Measurement Using 64Cu-ATSM PET/MRI: A Quantitative PET and MRI Study
Source: Diagnostics (Basel). 2023 May 10;13(10):1685. doi: 10.3390/diagnostics13101685 (PMC10216934; doi:10.3390/diagnostics13101685)
Supplement: Supplementary file 1 [file diagnostics-13-01685-s001.zip › diagnostics-2304974-supplementary.pptx]

## Slide 1
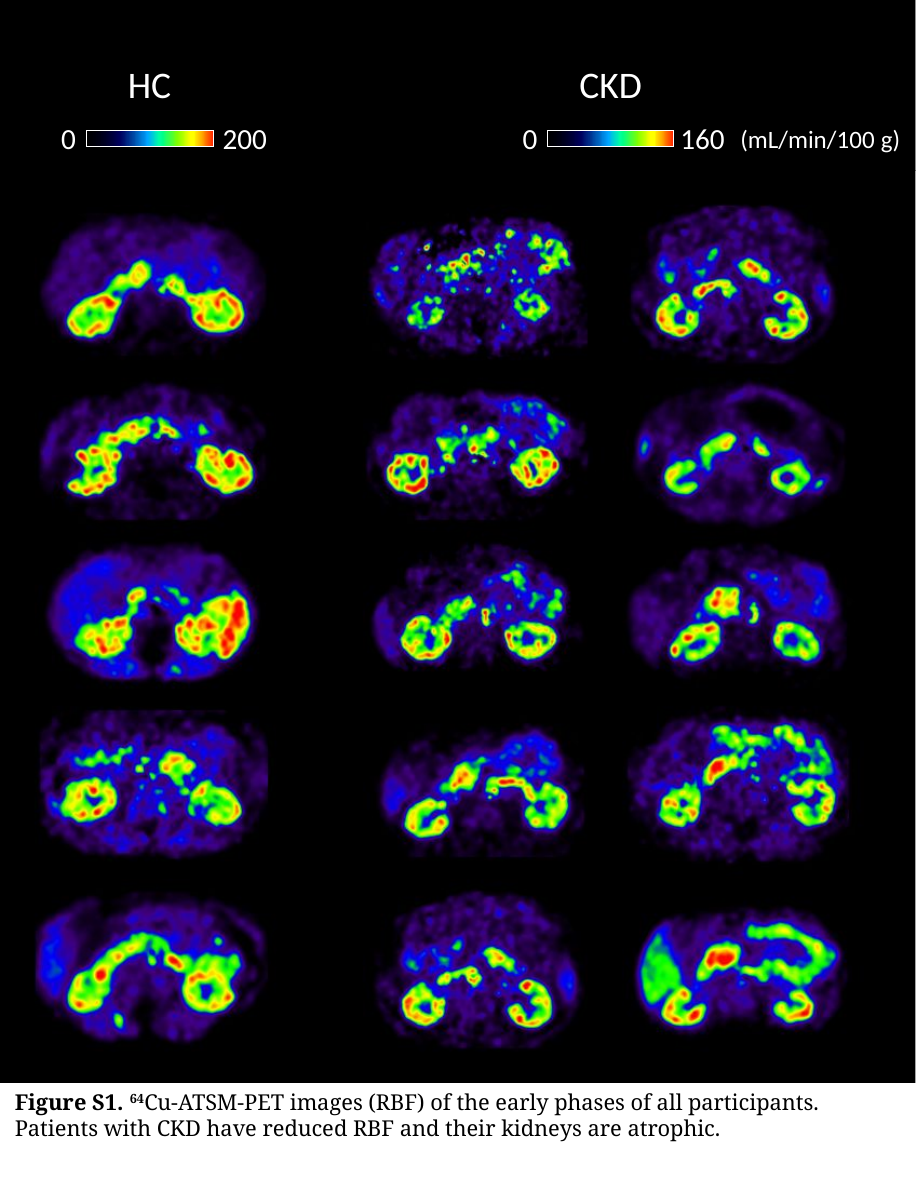

HC
CKD
0
200
0
160
(mL/min/100 g)
Figure S1. 64Cu-ATSM-PET images (RBF) of the early phases of all participants. Patients with CKD have reduced RBF and their kidneys are atrophic.
